# Acute type A aortic dissection repair in an octogenarian with achondroplasia: a case report

**DOI:** 10.1186/s40792-018-0461-0

**Published:** 2018-06-08

**Authors:** Shuji Moriyama, Masahiko Hara, Yasushi Kaneko

**Affiliations:** Department of Cardiovascular Surgery, Kumamoto Rosai Hospital, 1670 Takehara, Yatsushiro, Kumamoto 866-8533 Japan

**Keywords:** Acute type A aortic dissection, Achondroplasia, Octogenarian

## Abstract

**Background:**

Achondroplasia is an inherited disorder and the most common type of short-limbed dwarfism in human beings, affecting more than 250,000 individuals worldwide. To the best of our knowledge, no study has reported a correlation between achondroplasia and aortic dissection. Here, we report a rare case of acute type A aortic dissection repair in a patient with achondroplasia.

**Case presentation:**

An 82-year-old Japanese female with achondroplasia was admitted to our hospital because of acute-onset severe chest pain migration to her back accompanied by numbness and pain in the right lower limb. A computed tomography scan revealed acute type A aortic dissection with right leg ischemia because of an occlusion of the right common iliac artery. We successfully performed hemiarch repair.

**Conclusions:**

This report presents the first case of a patient at such an advanced age with dwarfism and cardiac surgery and the second case to illustrate successful acute aortic dissection repair in achondroplasia. Of note, all procedures were performed without specialized equipment. Overall, this report adds to the experience of successful cardiac surgery in this unique patient population.

## Background

Achondroplasia is a genetic disorder that results in dwarfism and is the most common type of short-limbed dwarfism in human beings, reportedly affecting more than 250,000 individuals worldwide [1, 2]. In addition, the incidence of achondroplasia is estimated to be 1 in 10,000–30,000 live births per year, affecting both males and females alike [1, 2]. To the best of our knowledge, no correlation exists between achondroplasia and aortic dissection. Here, we report the first case of such an advanced-aged patient with dwarfism and cardiac surgery and the second case of successful acute aortic dissection repair in patients with achondroplasia in the English literatures [3–11].

## Case presentation

**Fig. 1:**
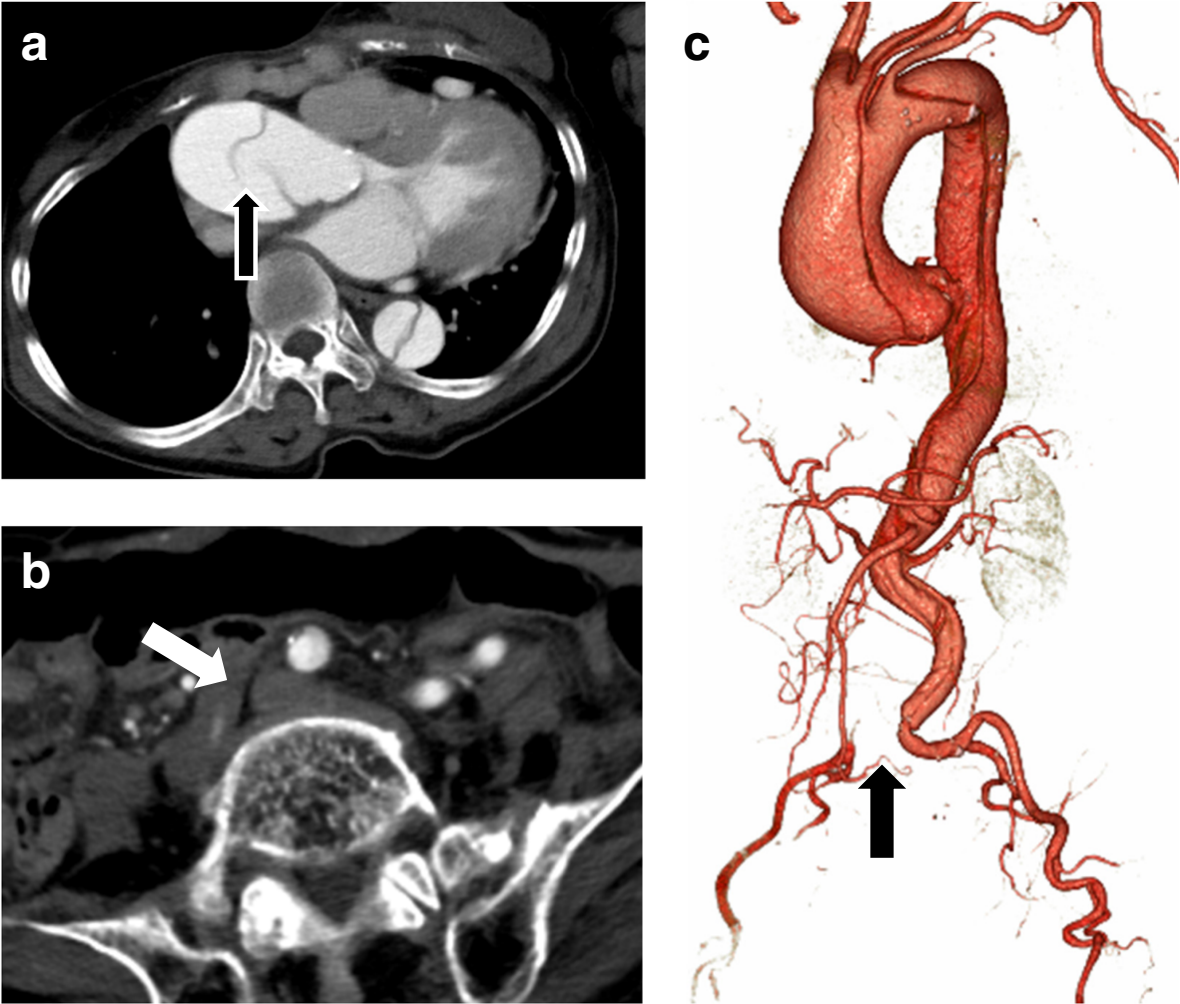
A preoperative CT scan showing acute type A aortic dissection. The dissection entry was found in the ascending aorta (**a** arrow). The true lumen of the right common iliac artery was compressed by the thrombosed false lumen (**b, c** arrow)

An 82-year-old Japanese female [height 100 cm, weight 27 kg, body surface area (BSA) 0.82 cm^2^] who was diagnosed with achondroplasia at birth based on the short stature and lateral curvature of the spine was admitted to our hospital because of acute-onset severe chest pain migration to her back accompanied by numbness and pain in the right lower limb. Her medical history was significant for achondroplasia, hypertension, and chronic obstructive pulmonary disease. A computed tomography (CT) scan revealed a dissection flap in the dilated ascending aorta that extended through the aortic arch to the bilateral iliac arteries (Fig. 1a). In addition, the great vessels of the aortic arch and the major abdominal branches communicated with the true lumen. However, an occlusion was reported in the right common iliac artery (Fig. 1b and c). Although electrocardiography revealed normal sinus rhythm with no apparent ST elevation, echocardiography revealed mild aortic valve regurgitation without left ventricular asynergy and pericardial effusion. Furthermore, laboratory findings revealed anemia (hemoglobin level, 8.1 g/dL) and hyperfibrinolysis (fibrin/fibrinogen degradation product level 67.6 μg/mL, d-dimer level 33.8 μg/mL). Based on these findings, the patient was diagnosed with acute type A aortic dissection with right limb ischemia because of right common iliac artery occlusion. Accordingly, we recommended repair of type A aortic dissection. However, we performed femorofemoral shunting before repair of type A aortic dissection to prevent complications of prolonged limb ischemia.

After cannulating the bilateral radial arteries under ultrasonographic guidance for monitoring of the arterial blood pressure, we induced general anesthesia and performed intubation with a 6.5-mm cuffed endotracheal tube using a video laryngoscope. We fixed the endotracheal tube at 19 cm after verifying the position with fiberoptic scope. Then, we cannulated the right jugular vein under ultrasonographic guidance for central venous access and pressure monitoring. Of note, narrow pharyngolarynx and short neck rendered transesophageal echocardiography impossible. We maintained anesthesia with propofol, rocuronium, and remifentanyl infusion.

Despite exposing common femoral arteries, these had a 9-mm diameter and could not be dissected, and the right femoral artery had no pulsatile flow. We antegradely cannulated a 4-French (Fr) sheath at the right common femoral artery. Then, we shunted from the right radial artery to the right femoral artery to ensure blood supply to the right lower limb and minimize ischemia-reperfusion injury until cardiopulmonary bypass (CPB) was established.

**Fig. 2:**
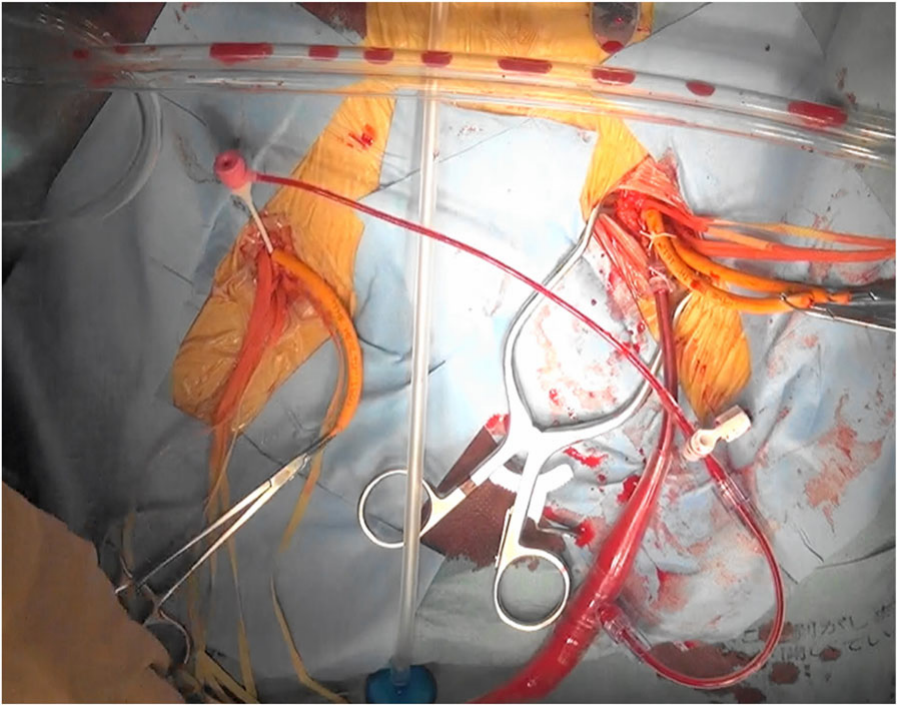
An intraoperative photograph showing the femorofemoral shunt with arterial cannula sidearm

**Fig. 3:**
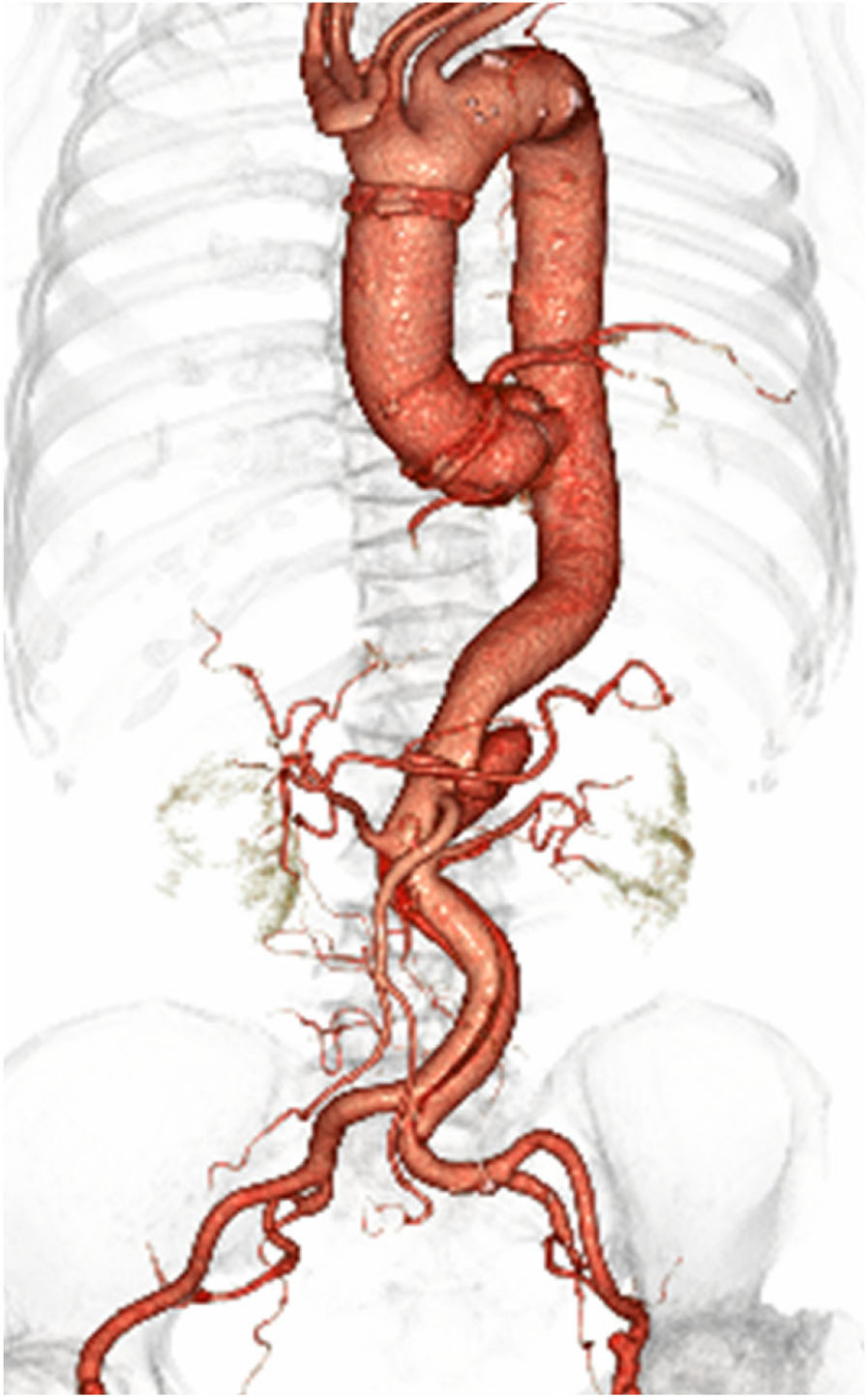
A postoperative 3D CT scan showing antegrade perfusion of the right common iliac artery. However, femorofemoral crossover bypass was occluded

**Fig. 4:**
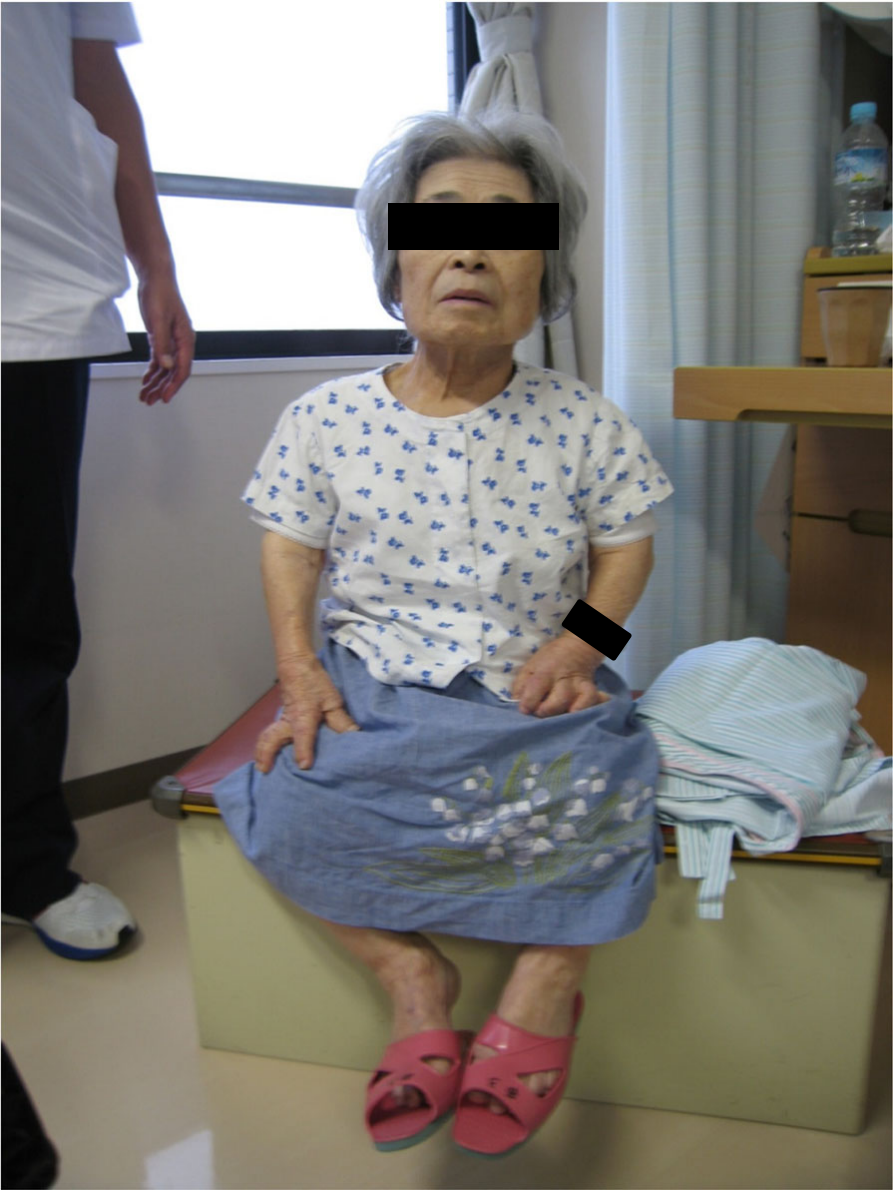
Our patient at 40 days after the operation

The chest was opened uneventfully through median sternotomy with relatively stable hemodynamics. The patient’s heart and lung were normal in size and appearance. After heparinization (300 IU/kg) and obtaining an activated clotting time (ACT) > 400 s, we initiated CPB using 16-Fr arterial cannula insertion from the left femoral artery and 28-Fr cannulas for the superior and inferior vena cava. After establishing CPB, we supplemented flow in the right lower extremity through the sheath from a sidearm of the left femoral arterial cannula and proceeded with hemiarch reconstruction (Fig. 2). CPB flow rates maintained a cardiac index of 2.0–2.5 L/min/m^2^. We placed a vent in the right upper pulmonary vein once the heart started to fibrillate, followed by hemoconcentration through the procedure. We used moderate systemic hypothermia to a target temperature of 27 °C. After initiating circulatory arrest at 27 °C rectal temperature, we initiated antegrade selective cerebral perfusion following the ascending aortotomy. Then, the dissection flap with a primary tear was identified in the mid-to-proximal ascending aorta. Hemiarch repair with a one-branched J Graft Shield Neo 26-mm Dacron graft (Japan Lifeline, Tokyo, Japan) was performed. Upon completion of the hemiarch repair, although the patient demonstrated the pulsatile flow in both femoral arteries, the low-flow state existed in the right lower extremity, leading us to perform a femorofemoral crossover bypass. The patient was shifted to the surgical intensive care unit in stable condition. Her immediate postoperative course remained uneventful, and she recovered without neurological or motor deficits in the bilateral lower extremities. A postoperative histological examination of the removed aortic wall revealed no specific findings, such as cystic medial necrosis or infiltration of the inflammatory cells, in an aortic wall. Postoperative CT scan revealed antegrade perfusion of the right common iliac artery; however, the femorofemoral crossover bypass was occluded (Fig. 3). Finally, on postoperative day 41, the patient was discharged to a geriatric health service facility (Fig. 4).

## Conclusions

Achondroplasia is a rare genetic bone growth disorder that results in disproportionate dwarfism and is characterized by good general health and average life expectancy. However, the average life expectancy of patients with achondroplasia has been reported to decrease by 15 years compared to healthy population [1]. To the best of our knowledge, no correlation exists between achondroplasia and cardiac or aortic disorder. To date, only nine cases of cardiac surgeries and dwarfism have been reported [3–11]. However, our case is the most advanced-age case of cardiac surgery among patients with achondroplasia and the second case to report dealing with an acute aortic dissection repair.

Studies have reported some problems while providing general anesthesia to patients with achondroplasia requiring surgery. For instance, the abnormal anatomy of the upper respiratory tract and skeletal abnormalities could pose complications in tracheal intubation. In addition, the guidelines for the endotracheal tube size selection for dwarfs remain unclear. Mayhew et al. suggested that the endotracheal tube size for patients with achondroplasia is predicted based on patient’s weight [12]. We used a smaller-sized endotracheal tube because the glottic opening of the patient was small. Despite preparations for difficult airway, the trachea was intubated uneventfully with a neutral neck position and a video laryngoscope. Furthermore, the length of the insertion tube was guided by a fiberoptic scope to avoid any endobronchial intubation.

Of note, predicting the cardiac output required to maintain tissue perfusion potentially during CPB is challenging. Usually, the flow on CPB is based on the cardiac index of 2.0–2.5 L/min/m^2^ using BSA computed by various formulas and nomograms. However, none of the formulas is validated for patients with achondroplasia. In addition, the size of various cannulas for conducting CPB remains unclear. To date, four case reports have described the size of the cannula in adult patients with achondroplasia [6–9]. Scafuri et al. used a pediatric cannula for aortic valve replacement with root enlargement in a 56-year-old woman with achondroplasia; however, they encountered no problem during CPB [6]. Other studies have reported using adult cannulas for the repair of type A aortic dissection [7], aortic valve replacement [8], and pulmonary endarterectomy [9]. Despite no consensus on this matter, an adult cannulation can often be performed successfully and should be considered given that these studies reportedly mentioned increasing their CPB flow to maintain adequate tissue perfusion. In our case, we adjusted the flow rate by monitoring the perfusion parameters such as lactate, SvO_2_, or cerebral rSO_2_. Overall, our patient responded well to CPB.

In conclusion, we reported the first case of a patient at such an advanced age with dwarfism and cardiac surgery and the second case of a successful acute aortic dissection repair in a patient with achondroplasia. Despite performing no comprehensive workup before the operation because of the urgency of the case, the procedures were uncomplicated. Furthermore, the procedures were performed without requiring specialized equipment. Hence, we believe that this report adds to the experience of successful cardiac surgery in this unique patient population.

## Abbreviations

ACT: Activated clotting time
BSA: Body surface aria
CT: Computed tomography
CPB: Cardiopulmonary bypass
